# Sentinel Lymph Node Biopsy in Melanoma: Overview and Updates

**DOI:** 10.3390/ijms26199469

**Published:** 2025-09-27

**Authors:** Adrian Mansini, Shah Aarohi, Mario Della Mura, Joana Sorino, Gerardo Cazzato, Alessio Giubellino

**Affiliations:** 1 Department of Dermatology, Rush University Medical Center, Chicago, IL 60607, USA; adrian_mansini@rush.edu; 2 Department of Laboratory Medicine and Pathology, University of Minnesota, Minneapolis, MN 55455, USA; shah0639@umn.edu; 3 Section of Molecular Pathology, Department of Precision and Regenerative Medicine and Ionian Area (DiMePRe-J), University of Bari Aldo Moro, 70121 Bari, Italy; mariodellamura1@gmail.com (M.D.M.); j.sorino@studenti.uniba.it (J.S.); gerardo.cazzato@uniba.it (G.C.); 4 Masonic Cancer Center, University of Minnesota, Minneapolis, MN 55455, USA

**Keywords:** melanoma, sentinel lymph node, biopsy, metastasis

## Abstract

The role of regional lymph nodes in melanoma metastasis has long been recognized. This review will detail the evolving role of sentinel lymph node biopsy (SLNB) in melanoma management in the era of adjuvant therapies. We analyze key themes and findings from recent publications, highlighting both areas of consensus and ongoing controversies. While the landscape of melanoma management continues to evolve with the advent of novel therapeutic combinations, SLNB remains a valuable tool for staging and potentially provides therapeutic benefit. However, optimal patient selection for SLNB requires careful consideration of individual risk factors and benefits of adjuvant therapy.

## 1. Introduction

### Background

The sentinel lymph node biopsy (SLNB) technique has emerged as a crucial method in the staging and management of melanoma, offering valuable insights into the metastatic potential of this aggressive skin cancer [1,2,3]. It is essential to identify the sentinel lymph nodes, which are the first nodes to receive drainage from a tumor site. This process typically involves the injection of a radioactive tracer and/or blue dye near the melanoma, allowing surgeons to visualize and subsequently remove these key lymph nodes for pathological examination. An accurate assessment of these nodes can significantly influence treatment decisions and prognostic evaluations, ultimately improving patient outcomes in melanoma management. The integration of SLNB into clinical practice has not only enhanced the precision of melanoma staging but also paved the way for personalized treatment strategies, enabling clinicians to tailor interventions based on individual patient risk profiles [2,4]. This approach has led to a more nuanced understanding of disease progression and recurrence, allowing for better monitoring and follow-up care tailored to each patient’s unique circumstances.

The advancements in cancer treatment, particularly the progress in tumor immunotherapy, represent a significant turning point that necessitates further exploration to fully understand the possibilities and challenges ahead [5]. Recent insights from cancer sequencing, clinical trials, and mechanistic studies, especially in immunotherapy, are paving the way for therapeutic improvements in treating both micro and macro-metastases. By uncovering the origins and characteristics of metastases, these findings reveal new avenues for developing more effective strategies to target metastatic relapse and enhance patient outcomes [5].

## 2. Sentinel Lymph Node Biopsy Techniques in Melanoma

### 2.1. Pre-Operative Lymphatic Mapping

Pre-operative lymphatic mapping is a critical step in sentinel lymph node biopsy for melanoma, facilitating the accurate identification and subsequent removal of sentinel lymph nodes, which are the first lymph nodes to receive lymphatic drainage from the primary tumor site [6]. This procedure traditionally involves the injection of a radiocolloid, often technetium-99m sulfur colloid, blue dyes, or a hybrid approach.

#### 2.1.1. Radiocolloid Injection

For many years, the standard practice has been using radiocolloids, such as technetium-99m sulfur colloid, for pre-operative lymphatic mapping [7,8]. The radiocolloid is typically injected intradermally around the primary melanoma site [6]. Following injection, the radiocolloid travels through the lymphatic channels to the regional lymph nodes, allowing for the identification of the sentinel lymph node(s) using a gamma camera. This allows for the accurate identification of the sentinel lymph node. The sentinel lymph node can then be targeted and surgically excised for pathological examination, providing valuable information about the potential spread of the melanoma.

Some studies in melanoma have also explored the use of lymphocele or lymphoscintigraphy, a specialized nuclear medicine imaging technique that generates visual representations of the lymphatic system. This technique allows visualization of the lymphatic drainage pattern prior to the surgical procedure [1,4,9]. However, no unique protocol has been established [9]. Recent studies have explored the use of different radiocolloid formulations and injection techniques to optimize the accuracy and efficiency of this approach. For example, some researchers have investigated the use of smaller particle-size radiocolloids, which may improve lymphatic drainage and provide more precise sentinel lymph node identification [10,11]. Furthermore, the injection technique has also been refined, with studies exploring intradermal, subdermal [12], and even intraoperative injection approaches, aiming to enhance the reliability and reproducibility of the pre-operative lymphatic mapping process.

#### 2.1.2. Lymphatic Mapping with Blue Dye

Blue dyes, such as isosulfan blue and methylene blue, are commonly used during surgery to visually identify the sentinel lymph node [1,13]. The dye is injected near the primary melanoma site, enabling the surgeon to trace the lymphatic channels leading to the sentinel lymph node. This visual cue complements the radiocolloid-based method, helping the surgeon accurately locate and remove the sentinel lymph node.

However, the use of blue dye alone has been associated with lower identification rates compared to the combined application of radiocolloid and blue dye [14]. Additionally, the blue dye method may be less effective in identifying smaller or deeper lymph nodes, and it has the potential to cause allergic reactions or skin discoloration [8,14].

Despite these drawbacks, blue dye offers the advantage of providing a direct visual marker, making it easier for the surgeon to identify the sentinel lymph node. Nevertheless, its efficacy is improved when combined with radiocolloid, or when used as part of a hybrid technique.

#### 2.1.3. Hybrid Imaging (SPECT/CT, PET/CT)

The combination of radiocolloid and blue dye, known as the “hybrid technique,” has emerged as a more comprehensive approach to pre-operative lymphatic mapping, and demonstrated improved identification rates of sentinel lymph nodes compared to either method alone. The hybrid technique leverages the strengths of both radiocolloid and blue dye to improve the accuracy and reliability of sentinel lymph node identification [1,15].

The hybrid technique involves injecting radiocolloid and blue dye around the primary melanoma site. The radiocolloid allows a gamma probe to detect the sentinel lymph node during surgery, while the blue dye provides a visual cue for the surgeon, facilitating the accurate localization and removal of the key lymph nodes for pathological examination [16]. Several studies have demonstrated that the hybrid technique can result in higher sentinel lymph node identification rates compared to either method alone [14,17]. Thus, the hybrid technique has become the preferred method for identifying sentinel lymph nodes.

Additionally, advances in imaging technology, such as single-photon emission computed tomography/computed tomography (SPECT/CT) and positron emission tomography/computed tomography (PET/CT), have further enhanced the pre-operative lymphatic mapping process.

These hybrid imaging modalities provide detailed anatomical and functional information, allowing for more precise identification of the sentinel lymph node prior to the surgical procedure. These advanced imaging techniques can help reduce the number of lymph nodes that need to be surgically removed, potentially decreasing the risk of complications and improving the overall patient experience.

### 2.2. Intraoperative SLN Identification and Excision

The intraoperative identification and excision of the sentinel lymph node are crucial steps in the sentinel lymph node biopsy procedure for melanoma. The use of a gamma probe, which detects the radioactive signal from the injected radiocolloid, has become a standard technique for intraoperatively identifying the sentinel lymph node. The visual cue provided by the blue dye also aids in identifying the sentinel lymph node during the surgical procedure.

#### 2.2.1. Gamma Probe Guidance

During the intraoperative phase, the sentinel lymph node is identified using a gamma probe. The gamma probe is used to detect the radioactive signal emitted by the radiocolloid that has accumulated in the sentinel lymph node, guiding the surgeon to the appropriate lymph node for removal. The use of the gamma probe has become a standard practice in sentinel lymph node biopsy, as it allows for the accurate identification and targeted excision of the sentinel lymph node.

#### 2.2.2. Visual Identification with Blue Dye

The use of blue dye, such as isosulfan blue or methylene blue, has been an integral part of the intraoperative identification of the sentinel lymph node. The blue dye is injected around the primary melanoma site, and the lymphatic channels leading to the sentinel lymph node become visually apparent during the surgical procedure.

The visual cue provided by the blue dye can aid the surgeon in identifying the sentinel lymph node, particularly in cases where the radiocolloid signal is weaker or more diffuse. However, using blue dye alone has been associated with lower identification rates compared to using radiocolloid or the hybrid technique [14].

#### 2.2.3. Number of SLNs to Remove

The quantification of sentinel lymph nodes designated for excision during surgical intervention has emerged as a subject of persistent scholarly debate [2,18,19]. Historically, the conventional methodology has entailed the resection of all discernible sentinel lymph nodes; however, contemporary research indicates that the surgical removal of a constrained quantity of sentinel lymph nodes may suffice in specific clinical scenarios, particularly when patients undergo adjunctive oncological therapies [20]. This evolution in surgical philosophy is underpinned by the acknowledgment that an extensive excision of lymph nodes correlates with heightened surgical morbidity, exemplified by lymphedema, without necessarily augmenting prognostic insights [2]. Consequently, there has been a discernible movement towards a more judicious and targeted excision of sentinel lymph nodes, concentrating on the detection and removal of pivotal lymph nodes that are most predisposed to containing metastatic pathology. This methodology harbors the potential to alleviate the overall surgical encumbrance on the patient while concurrently furnishing requisite data for precise pathological staging and therapeutic strategies.

In the context of melanoma, the quantity of sentinel lymph nodes (SLNs) excised during sentinel lymph node biopsy (SLNB) exhibits variability contingent upon the characteristics of the patient and the tumor in question. Empirical investigations have demonstrated that the resection of up to five SLNs can yield a recovery rate exceeding 99% of positive SLNs in individuals diagnosed with breast cancer, with the total number ranging from two to ten based on distinct variables [21]. Although analogous data pertaining to melanoma remains scarce, the foundational principles governing SLNB in melanoma bear resemblance to those applicable to breast cancer. Determinants that affect the number of SLNs excised encompass tumor dimensions, anatomical location, and the surgical approach adopted by the practitioner. The decision-making process is meticulously customized to accommodate the distinctive circumstances of each patient, striving to achieve a balance between precise staging and the mitigation of surgical morbidity.

### 2.3. Pathological Examination of the SLN

The pathological examination of the sentinel lymph node is a crucial step in the sentinel lymph node biopsy procedure for melanoma. Over the past five years, there have been advancements in the processing techniques, immunohistochemical staining, and molecular techniques used to assess the presence and extent of metastatic disease in the sentinel lymph node. The incorporation of these advanced processing, immunohistochemical, and molecular techniques in the pathological examination of the sentinel lymph node has contributed to more accurate and sensitive detection of metastatic disease in melanoma patients.

#### 2.3.1. Processing Techniques

The processing techniques employed in the pathological examination of the sentinel lymph node have evolved to provide more detailed and accurate information [22,23]. These techniques include serial sectioning, which involves cutting the lymph node into thin slices for a more comprehensive examination, and immunohistochemical staining. The use of serial sectioning allows for a detailed, layer-by-layer analysis of the lymph node, increasing the chances of detecting any metastatic disease that may be present [22,24]. Similarly, immunohistochemical staining techniques utilize specific antibodies to identify the presence of melanoma cells, even in cases where they are not readily visible through standard histological staining methods. The use of these advanced processing techniques has improved the sensitivity of the detection of metastatic disease within the sentinel lymph node, which can have important implications for the staging and treatment of melanoma patients [22,23].

#### 2.3.2. Immunohistochemical Staining

Immunohistochemical staining is a crucial component of the pathological examination of the sentinel lymph node. Immunohistochemistry helps in identifying melanoma cells that may not be apparent using standard histology, enhancing the accuracy of staging and treatment decisions [25,26,27]. This technique utilizes antibodies that bind to specific proteins or markers present in melanoma cells, enabling the detection of metastatic disease that may not be visible on routine histological examination. The application of immunohistochemical staining has become more widespread, as it has been shown to significantly improve the sensitivity and accuracy of the pathological assessment of the sentinel lymph node [28]. This technique can provide valuable additional information to complement the traditional histopathological evaluation, ultimately leading to more accurate staging and informing the development of personalized treatment strategies for melanoma patients.

The most common immunohistochemical stains used to evaluate metastatic melanoma cells are S100, HMB-45, and Melan-A [29,30]. The S100 protein is found in melanoma cells, melanocytes, nodal nevus cells, Langerhans cells, chondrocytes, adipocytes, and Schwann cells; therefore, it is considered a less specific marker as it is expressed in a variety of normal cell types. HMB-45 demonstrates reactivity to melanocytes and can serve as a useful marker for melanoma [25]. Melan-A, also known as MART-1, is a protein expressed by melanocytes and melanoma cells. Melan-A/MART-1 has demonstrated superior performance in identifying and diagnosing micrometastases within sentinel lymph nodes in cutaneous melanoma. Melan-A exhibits greater sensitivity compared to HMB-45, but it may also stain nodal nevi [31,32]. Conversely, S-100 can be more challenging to interpret and exhibits lower diagnostic accuracy [33]. The European Organization for Research and Treatment of Cancer guidelines recommend utilizing the S100 marker primarily during the processing of sentinel lymph nodes, as S100 is expressed in most melanoma cases [31]. However, given the limitations of individual markers, a combination of immunohistochemical stains is necessary to comprehensively evaluate potential melanoma metastases.

The application of immunohistochemical staining has significantly improved the accuracy of sentinel lymph node staging, enabling clinicians to make more informed decisions regarding adjuvant therapy and follow-up strategies. The proper interpretation requires careful consideration of staining patterns, cellular morphology, and the clinical context.

#### 2.3.3. Molecular Techniques

Molecular techniques, such as polymerase chain reaction (PCR), have increasingly gained attention in the pathological evaluation of sentinel lymph nodes in melanoma patients [23,34,35]. These advanced methods enable the detection of specific genetic or molecular markers associated with melanoma, even in cases where metastasis may not be evident on routine histological examination using standard hematoxylin and eosin staining. The use of molecular techniques, including PCR-based assays, has the potential to enhance both the sensitivity and specificity of sentinel lymph node assessments, providing valuable supplementary information alongside traditional histopathological evaluations. This improved sensitivity can contribute to more accurate staging, which in turn aids in the development of personalized treatment strategies for melanoma patients [35,36].

Furthermore, PCR-based assays in sentinel lymph node biopsy (SLNB) can help assess the degree of immune dysfunction in the sentinel lymph nodes of melanoma patients. For instance, Lee et al. employed quantitative real-time PCR to analyze gene expression related to various cytokines and immunosuppressive cell surrogates in the lymph nodes of melanoma patients. Their findings revealed a correlation between sentinel lymph node tumor burden and the degree of immune dysfunction within the SLNs [37]. This suggests that tumor burden in the sentinel lymph node may be linked to an immunosuppressive microenvironment that facilitates tumor growth and metastasis.

### 2.4. Summary

The sentinel lymph node biopsy technique has become a widely accepted and valuable tool in the management of melanoma patients. Over the past five years, significant advancements have been made in the various aspects of this procedure, including pre-operative lymphatic mapping, intraoperative identification and excision of the sentinel lymph node, and pathological examination of the removed lymph node.

The use of radiocolloid, blue dye, and hybrid imaging techniques in the pre-operative lymphatic mapping stage has improved the accuracy and reliability of the process, ensuring that the appropriate lymph nodes are identified for excision. These techniques have become increasingly sophisticated, allowing for more precise and comprehensive mapping of the lymphatic system [38], which is crucial for successful sentinel lymph node biopsy.

In the intraoperative stage, the use of gamma probe guidance, visual identification with blue dye, and a targeted approach to the number of sentinel lymph nodes removed have contributed to more efficient and effective surgical procedures. This targeted approach has the potential to reduce the surgical burden on patients while still providing the necessary information for accurate staging and treatment planning [38].

Finally, advancements in processing techniques, immunohistochemical staining, and molecular techniques employed in the pathological examination of the sentinel lymph node have led to more sensitive and accurate detection of metastatic disease. These advanced techniques, such as serial sectioning, immunohistochemical staining, and PCR-based assays, have the potential to further enhance the sensitivity and specificity of the pathological assessment, ultimately leading to more accurate staging and informing the development of personalized treatment strategies for melanoma patients.

As our understanding of melanoma biology and the role of sentinel lymph node biopsy continues to evolve, we can expect further refinement and improvements in this technique, which will enhance patient outcomes and quality of life.

## 3. Clinical Significance of SLNB

### 3.1. The Clinical and Prognostic Significance of SLNB: From Origins to Present Day

The initial metastatic pathway for many malignancies, including melanoma, is via lymphatic dissemination to regional lymph nodes. Recognized over a century ago, this observation has stimulated interest in optimizing both lymph node assessment and treatment [39]; the latter may yield accurate staging and prognostic information and potentially remove early metastases interrupting the neoplastic spread and improving patient survival. Across multiple cancer types, there has been a longstanding debate regarding the clinical significance of lymphatic spread, particularly with respect to the role of regional nodal surgery at initial presentation, even in the absence of clinically apparent nodal involvement. Melanoma was among the earliest malignancies in which this controversy was systematically investigated [40]. Historically, pathological evaluation of regional lymph nodes required complete nodal basin removal, a procedure associated with consistent perioperative and long-term morbidity. This strategy, referred to as elective lymph node dissection (ELND), was controversial for over a century. While ELND was evidently beneficial in terms of survival for patients with occult microscopic nodal metastases detected through early surgery, those with clinically apparent nodal progression fared worse [41]. However, most melanoma patients present without nodal metastases and ELND confers no therapeutic benefit, but only risks such as infection, seroma formation, wound healing complications, nerve injury, and lymphedema [39]. The technique now known as SNLB was pioneered by Morton and colleagues at the University of California, Los Angeles (UCLA) in the 1970s and 1980s [42], based on lymphatic physiology. In fact, as tracers and imaging advanced, it became evident that lymphatic drainage of a circumscribed anatomical region is often directed to one or a small number of nodes, rather than the entire basin. Consequently, those specific nodes, the SNLs, were hypothesized to reflect the status of the entire nodal basin: if negative, the remainder would likely be disease-free. SNLB thus enabled targeted identification and pathological examination of the lymph nodes draining the primary tumor, allowing patients with negative SNLB to avoid the more extensive and morbid full dissection. In addition, SNLB permits more detailed pathological scrutiny, as it represents a smaller tissue sample [39]. Overall, this transition from ELND to SLNB introduced a minimally invasive alternative for regional nodal assessment.

This paradigm shift was rigorously evaluated in the Multicenter Selective Lymphadenectomy Trial I (MSLT-I) [43], a prospective randomized international trial involving centers in North America, Australia, Europe, and Israel. The primary analysis focused on patients with intermediate-thickness melanomas (1.2–3.5 mm), who were most likely to benefit. Participants were randomized to wide local excision (WLE) plus SNLB, with complete (regional) lymph node dissection (CLND) if SNLB-positive, versus WLE alone, with CLND only upon clinical nodal recurrence. Both arms showed a similar cumulative incidence of regional nodal involvement (16–17%). SLN status emerged as one of the strongest prognostic indicators (HR for melanoma-specific mortality 2.40; 95% CI 1.61–3.56; *p* < 0.001), reinforcing existing retrospective findings. Notably, SLNB conferred a significant recurrence-free survival (RFS) benefit in both intermediate-thickness and thick melanomas. In subgroup analyses among patients with nodal involvement (either SLN-positive or node-detected on observation), earlier surgical removal via SNLB conferred a survival advantage (HR 0.56; 95% CI 0.37–0.84; *p* = 0.006). This comparison suggests a clinical advantage for patients with intermediate thickness melanomas who harbor clinically occult nodal metastases and benefit from having that disease removed earlier rather than later. Regardless of any effect on overall survival (OS), MSLT-I confirmed the prognostic value of SLNB and led to its widespread adoption in melanoma management guidelines. Moreover, a meta-analysis of over 25,000 patients estimated a <5% nodal recurrence risk after a negative SLNB [44].

Consequently, based on the MSLT-I trial, every patient with SLN metastasis underwent completion dissection in that basin. Over time, it became evident that most SLN-positive patients harbored no additional disease in the remaining lymph nodes, raising questions about the necessity of routine CLND [44]. Two large, randomized trials, Multicenter Selective Lymphadenectomy Trial (MSLT-II) [19] and the German Cooperative Oncology Group selective node dissection trial (DeCOG-SLT) [45], addressed this issue. Both trials randomized SLN-positive patients to CLND or nodal observation. MSLT-II enrolled 1939 patients and evaluated melanoma-specific survival; DeCOG-SLT included 483 patients and assessed distant metastasis-free survival. The key findings of the major randomized clinical trials evaluating SLNB in melanoma are summarized in Table 1. Neither trial demonstrated a significant survival benefit from CLND; only disease-free survival, and specifically reduced nodal relapse, was improved in the dissection arms. Consequently, clinical practice shifted: observation with regular nodal ultrasound became the standard of care for most SLN-positive patients, without adjuvant CLND [46]. As a result, a positive SLNB was no longer an absolute indication for a CLND. Based on these pivotal trials, current guidelines do not recommend routine CLND after a positive SLNB. Instead, nodal observation with ultrasound and adjuvant systemic therapy are considered standard of care.

Nonetheless, precise nodal staging remains critical. SNLB provides vital prognostic information that guides the use of intensified surveillance, adjuvant systemic therapy, and clinical trial eligibility. It is now firmly established as the principal staging procedure in melanoma, incorporated into the AJCC TNM staging system [40]. Current consensus recommends SNLB should be based on individual risk assessments for nodal metastasis [47] and it is usually performed when the estimated risk exceeds 10%. It is generally not recommended when risk is <5%, with shared decision-making advised for risk between 5 and 10%. Risk estimation is facilitated by validated nomograms such as those from the Melanoma Institute Australia [48,49].

Considerations for follow-up vary accordingly. For instance, a patient with a T2a primary melanoma and a negative SLN may require only minimal follow-up without imaging, whereas a positive SLN warrants more intensive surveillance including nodal-basin ultrasound [19].

Regarding adjuvant treatment, there are now several studies that provide strong evidence for improved outcomes with adjuvant systemic therapy for stage III melanoma (pT1–4 N1), including RFS, distant metastasis-free survival, and OS, with immunotherapy and targeted agents. These studies provide a strong rationale for performing SLNB, as patients must be accurately staged to benefit from systemic therapy [50,51,52,53,54,55,56,57,58].

### 3.2. The Therapeutic Significance of SLNB: Evidence or Speculation?

The potential therapeutic impact of SNLB has been a subject of ongoing debate. Beyond its widely recognized prognostic value, SLNB also demonstrates therapeutic benefit. Although the latter is primarily driven by a reduction in nodal recurrence, its ability to improve regional disease control has clear clinical significance. The MSLT-I trial demonstrated excellent regional control in patients who underwent SLNB followed by CLND [43]. However, subsequent findings from the MSLT-II trial suggested that most of the therapeutic benefit could be achieved with SLNB alone. Specifically, more than 80% of SLN-positive patients who did not undergo CLND achieved durable disease control and did not develop nodal recurrence. This strongly supports the notion that SLNB alone can achieve definitive clearance of the nodal basin in most patients. In other words, SLNB itself removed all detectable nodal disease in most cases, thereby obviating the need for a more extensive and morbid dissection. These findings reinforce the concept that SLNB should be considered to have therapeutic, not merely prognostic, value [59].

Nevertheless, conceptual and methodological challenges remain when comparing outcomes between patients with SLN-positive disease detected early and those who develop nodal recurrence after a period of observation. Such comparisons inherently assume that all abnormal cells detected in sentinel nodes would inevitably progress to clinically significant disease. Many of these cells may be eliminated by host immune responses or fail to survive the inhospitable conditions of the lymphatic and circulatory systems. Therefore, a subset of SLN-positive findings may be biologically insignificant or “false positives.” In these cases, patients are inadvertently upstaged and may undergo unnecessary surgery or adjuvant therapy without clear clinical benefit [60].

Overall, while there is unequivocal evidence that SNLB improves RFS, its effect on melanoma-specific survival and OS remains less established. To date, no individual randomized clinical trial has demonstrated a definitive survival benefit from SLNB. Consequently, many have concluded that such a benefit may not exist. However, the available trials have been insufficiently powered to definitively resolve this question, and meta-analyses have yielded conflicting results. Thus, current evidence is inadequate to confirm or refute a survival advantage attributable to SNLB. If a survival benefit exists, it likely applies only to patients with nodal metastases and is expected to be modest, potentially translating into an absolute OS improvement of 3–4%. Given that this potential benefit appears to be achievable through SLNB alone, without the need for CLND, the risk-benefit profile remains acceptable for most patients [39].

### 3.3. SLNB in Specific Melanoma Subtypes

#### 3.3.1. Desmoplastic Melanoma

Desmoplastic melanoma (DM) represents a rare subtype of cutaneous melanoma (CM), accounting for approximately 4% of all cases [61,62,63]. It typically arises in sun-exposed areas of older male patients and is histologically distinguished by spindle-shaped malignant melanocytes set within dense collagenous stroma [61,63,64]. Despite its high rate of local recurrence, DM exhibits a relatively low incidence of lymph node metastasis compared to other CM variants, prompting debate over the role of SLNB in its staging [61,63,65].

DM is further subclassified into pure (PDM) and mixed (MDM) variants based on the proportion of desmoplastic features, with emerging evidence underscoring their prognostic divergence [66]. Indeed, MDM has been consistently linked to higher SLNB positivity (14.8% vs. 4.9%) and worse outcomes in terms of recurrence-free and disease-free survival when compared to PDM [61,63,67,68]. These findings are clinically relevant considering current SLNB recommendations, which suggest the procedure when the predicted positivity rate exceeds 10% [69].

Taken together, these data support consideration of SLNB, particularly in MDM cases, where the risk–benefit profile appears favorable. Still, further research is warranted to refine patient selection criteria and improve staging accuracy, emphasizing the need for consistent histopathologic classification, with particular emphasis on clearly specifying the DM subtype in pathology reports. Additionally, interobserver variability remains a relevant concern and should be addressed to ensure consistent and accurate classification.

#### 3.3.2. Acral Melanoma

Acral melanoma (AL) accounts for approximately 2–3% of all CM and is more commonly observed among Asian, African, and South American populations. It typically arises in glabrous acral skin, including the palms, soles, digits, and nail apparatus. Among its histologic subtypes, acral lentiginous melanoma (ALM) is the most frequent, while nodular and superficial spreading variants are less common [70,71]. AL is often diagnosed at more advanced stages, typically presenting with greater Breslow thickness and a higher incidence of ulceration, both of which contribute to its poorer prognosis. Interestingly, patterns of metastatic spread appear to vary according to pigmentation, with heavily pigmented lesions more commonly linked to initial regional lymph node involvement [72,73]. Focusing on lymphatic dissemination, recent evidence has shown that ALM is independently associated with a significantly increased risk of SLNB positivity, even after adjusting for established histopathologic predictors such as Breslow depth, ulceration, and mitotic index [74,75,76,77,78,79]. SLNB positivity rates in ALM reach 18.39% in clinical stage IB and 39.53% in stage II, exceeding the thresholds outlined by current National Comprehensive Cancer Network (NCCN) guidelines [80]. Additionally, an analysis from the SEER (Surveillance, Epidemiology, and End Results) registry reported an overall SLNB positivity rate of 25.7% [81,82], while a retrospective analysis involving 1764 patients with stage IB–IIC ALM demonstrated a 35% SLNB positivity rate [83]. Similarly, a Brazilian cohort reported a 34.2% positivity rate, with SLNB-positive patients exhibiting significantly worse DFS (33.8% vs. 46.7%, *p* = 0.001).

Taken together, these findings underscore the clinical utility of SLNB in ALM patients from stage IB onwards and suggest that standard prognostic algorithms developed for other CM subtypes may not be fully applicable to ALM, due to its distinct biological behavior. ALM is characterized by a unique mutational profile, frequent KIT and CDK mutations and rare BRAF alterations, as well as atypical metastatic patterns [84,85,86,87]. It may spread through non-sequential pathways, reducing the reliability of standard histopathologic predictors and highlighting the need for tailored staging and treatment strategies. However, further prospective studies are needed to better inform optimal management approaches for this distinct melanoma subtype.

#### 3.3.3. Mucosal Melanoma

Mucosal melanomas represent a significant diagnostic and management challenge, and the role of SLNB in this context remains poorly defined. Among them, anorectal melanoma (ARM) is a rare yet particularly aggressive subtype, with a markedly poorer prognosis compared to CM. The 5-year survival rate is approximately 20–25% (compared to 80% in CM), primarily due to delayed diagnosis, which occurs in two-thirds of patients presenting at advanced stages [88]. While lymphatic spread is common, lymphadenectomy has not shown a clear survival benefit [89,90]. In this context, the role of SLNB remains unclear due to limited evidence [91,92,93,94]. However, a recent study by Mistrangelo et al. [95] reported 100% identification and positivity rates in five patients undergoing inguinal SLNB, with three cases (60%) upstaged and redirected to adjuvant immunotherapy. Although the sample size was limited and follow-up was short, these findings suggest that SLNB could represent a valuable tool for more accurate staging in ARM. Beyond improving patient selection for adjuvant immunotherapy, SLNB may help avoid invasive lymphadenectomies and their associated morbidity, without compromising survival outcomes in patients with advanced ARM.

Vulvar melanoma (VM) is also rare, accounting for approximately 1% of all melanomas diagnosed in women and characterized by a particularly aggressive biological behavior, likely due to its mucosal origin [96,97,98]. It is associated with a poor prognosis, with a 5-year survival rate of approximately 45% [99]. To date, SLNB remains markedly underutilized: recent data show that only about 30% of patients undergo SLNB, with many still receiving no nodal evaluation at all. This limited use reflects the lack of dedicated guidelines due to the rarity of the disease, which often leads to variability in clinical practice and suboptimal staging [96].

Conjunctival melanoma (CjM) is a rare malignant neoplasm of the eye, with an estimated incidence ranging from 0.2 to 0.7 per 1,000,000 individuals [100,101]. Unlike uveal melanoma, CjM predominantly metastasizes via the lymphatic system, most commonly involving the preauricular, submandibular, and cervical lymph nodes [102]. SLNB is generally recommended when at least two of the following risk factors are present: non-limbal tumor location, tumor thickness greater than 2 mm, presence of ulceration, and a mitotic rate exceeding one figure per mm^2^. Reported SLNB positivity rates in the literature vary between 11% and 17%, while 5-year survival rates are estimated between 74% and 86% [103,104,105].

Recently, the successful use of programmed cell death protein 1 (PD-1) inhibitors has been described in cases of metastatic CjM [106]. Based on this, it has been suggested that patients with a positive SLNB, who are at increased risk of disease progression, might benefit from adjuvant systemic therapies [100]. Nevertheless, the overall utility of SLNB in CjM remains a matter of debate, primarily due to the rarity of the disease and the relatively low incidence of sentinel node positivity [102,103,107,108].

Melanoma in the oral cavity refers to a rare, aggressive malignant neoplasm arising from the melanocytes located within the mucosal lining of the mouth [109]. The etiology is unknown and the pathogenesis is poorly understood. Unlike cutaneous melanoma, oral mucosal melanoma typically presents in older adults and is often asymptomatic in early stages, which can delay diagnosis [110]. In oral mucosal sites, SLNB is associated with improved disease-free survival and distance metastases survival, reduced regional lymph node metastases, and led to a better trend toward overall survival [111]. Similarly to CjM, adjuvant therapy, especially with anti-PD-1 agents, improves survival outcomes. The combination of SLNB for staging and adjuvant therapy for systemic control is currently optimal, as adjuvant therapy alone does not replace the regional disease control provided by SLNB [46,112].

#### 3.3.4. Melanocytic Tumors of Uncertain Malignant Potential (MelTUMPs)

MelTUMPs comprise a heterogeneous group of melanocytic lesions with ambiguous biological behavior, which do not meet the criteria for a benign nevus but also fall short of the histopathologic thresholds required for a definitive melanoma diagnosis [113,114]. clinical management, particularly regarding the use of SLNB, remains controversial. According to Varey et al. [115], only 43% of patients with MelTUMPs underwent SLNB, with a pooled positivity rate of 32%—a substantial figure considering their borderline nature. Moreover, SLNB positivity rates were notably higher in younger patients (53% in <18 years, 25% in 18–40 years, and 11% in >40 years) and varied by histologic subtype (44% in pigmented epithelioid melanocytoma and 30% in atypical Spitz tumors), suggesting that age and histology may influence nodal involvement risk. However, only 6% of SLNB-positive patients experienced disease progression, complicating the interpretation of SLNB utility in this population [115].

SLNB indications remain ill-defined, with patient selection often relying on subjective clinical judgment, potentially leading to inflated positivity rates in selected cohorts. Despite frequent reporting of histopathologic features, their correlation with outcomes is inconsistent due to limited individual-level data. Moreover, the short median follow-up (<3 years) across studies may underestimate recurrence rates. Significant heterogeneity in diagnostic criteria and subtype classification further limits generalizability. Ultimately, the low rate of disease progression among SLNB-positive patients supports a cautious, individualized approach rather than routine SLNB in MelTUMPs [115].

#### 3.3.5. SLNB in In-Transit Melanoma Recurrences

In-transit melanoma recurrences occur in 5–10% of primary cutaneous melanomas and represent a distinct pattern of regional disease, characterized by dermal or subcutaneous metastases between the primary tumor site and the regional lymph node basin [116]. The role of SLNB in this setting is less clearly defined compared to primary cutaneous melanoma. However, several studies have demonstrated that in-transit lesions may still drain to sentinel lymph nodes, and SLNB can provide valuable prognostic information regarding further nodal involvement [117,118,119]. While SLNB is not routinely recommended for all in-transit recurrences, it may be considered in selected patients, particularly when accurate staging will influence decisions regarding adjuvant systemic therapy or clinical trial eligibility. Thus, SLNB remains a potentially informative staging tool in this subgroup, complementing imaging and clinical assessment to refine prognostication and therapeutic planning.

### 3.4. SLNB in Patients with Complete Lymph Node Dissection

In a subset of patients, melanoma may develop either as a second primary lesion or as a local recurrence in a region previously subjected to complete lymph node dissection (CLND) for a prior malignancy. In such cases residual lymph nodes are not typically expected to be present [120]. In the absence of established guidelines, these patients are often excluded from further nodal staging. This scenario presents a diagnostic and therapeutic dilemma, particularly considering that previous complete lymphadenectomy may disrupt normal lymphatic drainage pathways, potentially resulting in lower sentinel lymph node identification rates [17].

However, primary tumors are known to induce lymph-angiogenesis through the production of lymphangiogenic factors, facilitating the development of new lymphatic channels [121,122]. This could support the rationale for considering SLNB even after CL, given that the removal of regional lymph nodes does not preclude tumor cells from accessing the lymphatic system and migrating [123]. Therefore, omitting SLNB in these patients may result in the loss of critical pathological information necessary for accurate staging, appropriate adjuvant treatment selection, and the optimization of therapeutic outcomes [124].

In this context, Wainstein et al. [120] evaluated the feasibility of SLNB in patients with previous CLND, demonstrating a 100% success rate in sentinel lymph node identification through the combined use of lymphoscintigraphy and vital blue dye. In this study, 50% of patients had micrometastases that were not detected by preoperative radiological imaging. Although based on a limited case series, these findings suggest that SLNB may not only be technically feasible but also clinically meaningful in patients with a history of CLND and could be considered on a case-by-case basis as part of accurate and individualized staging, pending further validation in larger prospective studies.

## 4. Current Controversies and Future Directions

### 4.1. SLNB in Thin Melanomas

The role of SLNB in the management of patients with very thin melanomas, commonly described as less than 0.8 millimeters (mm) in Breslow thickness, remains an ongoing debate in the current literature. Current guideline recommendations for SLNB, based on Breslow thickness and ulceration, are summarized in Table 2. While SLNB has become a standard component of staging and prognostic significance in patients with intermediate to thick cutaneous melanomas [125], its utility in thin lesions remains unclear. Various literature suggests that the risk of nodal metastasis in this subset of patients is sufficiently low to justify omitting the procedure, particularly given the unclear therapeutic relevance of a positive SLNB in this context [126,127]. However, others suggest that specific criteria, including high-risk features such as patients age ≤ 45, mitotic rate > 1 mm^2^, and/or with ulceration [128], warrant the consideration of SLNB even in lesions under the 0.8 mm threshold [128,129,130]. In these cases, proponents explain that patients with these high-risk features should be informed of available evidence indicating a greater risk of nodal metastasis at the time of the initial diagnosis, meaning they may be more likely to benefit from SLNB [131,132]. This ongoing discourse highlights the importance of ongoing investigation to clarify risk stratification and optimize patient care for individuals with thin melanomas.

### 4.2. Alternatives to SLNB

Non-invasive techniques for lymph node staging encompass advanced imaging modalities and biomarker analyses, aimed at accurately detecting lymph node metastasis without the need for surgical intervention.

#### 4.2.1. Imaging Modalities

Ultrasound (US) remains a highly convenient, low-cost modality due to its accessibility and real-time imaging capabilities. Recent innovations, such as contrast-enhanced ultrasound and ultrasound molecular imaging (USMI) have increased the specificity of the US for lymph node staging [21]. Early manifestations of malignancy, such as asymmetric thickening and focal lobulation of the lymph node cortex, may be detected, as well as late-stage manifestations, including cortical thickening [133].

Magnetic Resonance Imaging (MRI), especially when enhanced with contrast agents, offers wonderful spatial resolution and tissue contrast, enabling the detection of metastatic lesions smaller than 1 mm. This quality enables the identification of micro metastases undetectable by conventional imaging techniques. The MRI is also recognized as having minimal biosafety concerns and is well-tolerated by patients [21]. However, its diagnostic accuracy is significantly reduced in non-enlarged lymph nodes, as size-based criteria are insufficient to distinguish benign from malignant lymph nodes [133,134]. This limitation is also noted in non-contrast Computed Tomography (CT), another form of cross-sectional imaging [133]. CT remains reliant on morphologic characteristics, including size, shape, and pattern, making it unreliable for early detection of lymph node metastasis.

Positron Emission Tomography (PET) and Single-Photon Emission Computed Tomography (SPECT) provide functional imaging capabilities that offer a high sensitivity, quantification imaging, and the option to do whole body scanning [21]. However, the inability to detect microscopic metastases limits their ability to be used as a modality for early nodal staging [133,134].

Fluorescence Molecular Imaging (FMI), although not invasive itself, is used to support intraoperative decision-making, enabling real-time visualization of metastatic lymph nodes during surgery. However, FMI is limited by its shallow tissue penetration, rendering it less effective for staging of deeper lymph nodes [21].

In photoacoustic imaging, the combination of optical and acoustic technology contributes to optimal biosafety and high-resolution, radiation-free imaging of superficial lymph nodes. However, penetration depth remains a significant barrier for detecting deep-seated metastatic nodes [21].

Together, these modalities offer a comprehensive spectrum of non-invasive options for staging of metastatic lymph nodes. Each with its own advantages and limitations, these technologies are continually being developed. None have fully replaced SLNB, yet emerging evidence supports their growing role in specific clinical contexts. Ongoing advancements will likely further refine their diagnostic utility.

#### 4.2.2. Biomarker-Based Techniques

In addition to imaging advancement, molecular biomarkers are used in the non-invasive staging of lymph node metastasis. Identification of reliable biomarkers for predicting lymph node metastasis in melanoma could potentially avoid the need for SLNB. By leveraging tumor-associated molecular signatures, biomarkers may reduce costs related to melanoma and minimize treatment-related morbidity [135].

Molecular imaging probes that target specific tumor biomarkers represent a promising non-invasive strategy for lymph node staging. Antibody-based probes, such as panitumumab-IRDye800CW directed at EGFR, have shown strong potential for detecting metastatic lymph nodes intraoperatively, especially in head and neck cancer [21]. Peptide-based tracers, including ^18^F-FP-R_0_1-MG-F_2_, offer advantages in biodistribution and clearance and have been used to highlight integrin α^v^β_6_-positive nodes on PET imaging [21]. In prostate cancer, small molecule agents such as ^68^Ga-PSMA have proven effective in identifying nodal disease with high sensitivity [21]. Finally, nanoparticle-based systems introduce further versatility by combining targeting ligands with imaging agents, allowing for both preoperative localization and live surgical navigation [21].

While many of these probes have been evaluated in other cancer types, their molecular pathways are relevant in the progression of melanoma. For instance, studies have identified vascular endothelial growth factors (VEGF), Tetraspanin CD9, and lymphatic vessel endothelial hyaluronan receptor-1 (LYVE-1) as immunohistochemical biomarkers predictive of SLNB status [135]. As research continues to map molecular signatures relevant to melanoma, targeted imaging probes may be used accordingly, leading to more patient-specific and less invasive nodal staging.

### 4.3. Molecular Profiling of Melanoma and SLN

Molecular profiling of primary melanomas and SLNs is an evolving strategy aimed at enhancing risk stratification and tailoring treatment in cutaneous melanoma. While histopathologic features such as Breslow thickness, ulceration, and mitotic rate remain foundational to staging, they may not fully capture the biological behavior of these tumors. Emerging molecular tools, such as liquid biopsy, microRNAs, and gene expression profiling (GEP), provide additional layers of prognostic insight that may refine clinical decision-making. GEP assays such as the 31-GEP (DecisionDx), CP-GEP (Merlin™), and 8-GEP (MelaGenix) analyze the activity of specific gene panels to classify tumors into risk categories [136,137,138]. For example, the i31-SLNB algorithm integrates clinicopathologic variables with the 31-GEP results to better predict SLN positivity, offering a more holistic risk assessment than histology alone [136]. However, a recent consensus statement from the Society of Surgical Oncology advises caution, explaining that GEP tests are not yet validated enough to replace SLNB or decisions regarding treatment outside the setting of clinical trials or specialized research [139].

Beyond the tumor, molecular staging of SLNs offers additional prognostic value. Literature has demonstrated that molecular analysis of SLNs using reverse transcriptase PCR could identify melanoma-specific transcripts in histologically negative nodes, and that patients who underwent completion of lymph node dissection based on positive molecular results experienced lower rates of regional recurrence [140]. These findings suggest that molecular profiling of SLNs may detect disease not captured by standard histopathology, informing patient decisions about surgical and systemic management.

The role of circulating molecular biomarkers is also being actively investigated. MicroRNAs (miRNAs), including miR-21, miR-155, and miR-10b, have been associated with SLN positivity and tumor progression. However, downregulation of tumor-suppressive miRNAs such as miR-125b and miR-203 is associated with a higher metastatic potential [136]. These blood-based biomarkers may offer a non-invasive method for preoperative risk stratification and surveillance. Additionally, expressions of lymphangiogenic markers such as VEGFR-3 and CD9 in primary tumors have been linked to SLN metastasis and may be incorporated into biomarker panels [135].

In conjunction with these techniques, the Cancer Genome Atlas has classified cutaneous melanoma into four molecular subtypes: BRAF, RAS, NF1, and Triple-Wild Type [141]. Each subtype has a distinct mutational signature, immune landscape, and implication for treatment response [141]. These mutations are not currently utilized to guide decisions related to an SLNB but represent critical aspects of molecular profiling and offer information related to tumor biology, progression, and therapeutic targeting.

Many of these tools are investigational, yet their potential to refine clinical decision-making related to SLNB is substantial. By integrating tumor biology with conventional staging, molecular profiling may help identify patients who could safely avoid SLNB. This incorporation may guide intensity of surveillance and select patients who are most likely to benefit from adjuvant therapy, thereby paving the way for more precise, personalized patient care.

### 4.4. Immunotherapy and SLNB

Management of regional lymph nodes in melanoma has significantly changed over the past several decades [142]. The emergence of adjuvant immunotherapy has reshaped the management of patients with melanoma who have a positive SLNB. Previously, a positive SLN often prompted an immediate complete lymph node dissection (CLND) to reduce regional recurrence risk [142]. However, with the availability of adjuvant molecular target therapy, the role of CLND has decreased, and the focus has shifted toward systemic control. Multiple pivotal studies have demonstrated that CLND does not improve overall survival in comparison to observation alone in patients with a positive SLNB [142,143,144]. This finding, combined with the development and success of adjuvant immunotherapy, has led to a shift in prioritizing systemic therapy over surgery for many patients [142,143,144].

Recent studies have confirmed that patients with a positive SLNB who forgo CLND will still receive excellent outcomes when treated with adjuvant immunotherapy [139,142,143]. Current guidelines support the omission of CLND if patients are monitored with close surveillance imaging and offered adjuvant systemic therapy. However, sentinel lymph node status remains crucial even in the modern adjuvant era, as SLN status continues to serve as a powerful prognostic marker and key determinant for adjuvant therapies [136,139].

Essentially, integration of effective adjuvant immunotherapies in SLN-positive patients has shifted the therapeutic standard from aggressive surgical management to systemic disease control. SLNB remains important for staging and guiding adjuvant treatment, while CLND is no longer routinely recommended in the context of positive SLNB if adjuvant systemic therapy and appropriate surveillance are employed.

### 4.5. Minimally Invasive SLNB Techniques

Minimally invasive approaches to SLNB have evolved in the hopes of maintaining oncologic precision while reducing surgical morbidity. Like other surgical procedures, SLNB may cause pain, swelling, and ecchymoses at the surgical site and increases the risk of infection [145]. Patients have also reported allergic reactions and lymphedema [145]. Recent technological advancements, including video-assisted and fluorescence-guided techniques, have created opportunities to perform SLNB with less disruption to surrounding tissues. Current literature emphasizes these techniques in breast cancer.

Video-assisted SLNB has been explored as a technique to decrease the invasiveness of nodal staging, particularly when combined with three-dimensional computed tomographic (3D-CT) [146]. 3D-CT provides detailed visualization of the lymphatics, guiding precise sentinel node localization. It has been demonstrated that SLNB can be safely and accurately performed with minimal aesthetic compromise using this approach [146].

Fluorescence-guided methods, such as those involving indocyanine green (ICG) and near-infrared imaging, have expanded on the possibilities for a minimally invasive SLNB. The use of ICG fluorescence allows for live mapping of lymphatic channels and sentinel nodes without reliance on radiocolloid tracers, leading to simplified logistics and decreased patient exposure to radiation [146,147]. Studies incorporating endoscopic or video-assisted platforms for an ICG-guided SLNB report high sentinel node identification rates and favorable postoperative outcomes [147,148]. However, in melanoma, ICG faces significant anatomical challenges not encountered in cancers, such as those affecting the breast. Melanoma patients, particularly those with tumors on the trunk, head and neck, or oral cavity, exhibit highly variable lymphatic drainage patterns involving multiple, deep, or small nodal basins [149,150]. ICG’s limited tissue penetration may miss these deeper nodes, a limitation less problematic in breast cancer where lymphatic drainage is more predictable and nodes are typically more superficial. Consequently, the standard of care in melanoma remains technetium-99m (Tc-99m) radiocolloid-based lymphoscintigraphy, with ICG serving as a valuable adjunct for enhanced intraoperative visualization rather than a replacement for established techniques [149,151,152,153].

Robotic-assisted approaches have also been proposed, emphasizing enhanced provider dexterity and three-dimensional visualization to access SLN with precision. However, the adoption of robotic SLNB is currently limited by factors including cost, lack of large-scale comparative study outcomes, and technological complexities [133].

Although promising, minimally invasive SLNB techniques face developmental challenges. Both specialized equipment and surgical expertise are required, and larger prospective studies are needed to validate long-term oncologic safety and cost in comparison to a conventional SLNB. Robust data in patients with melanoma is still required. This topic represents an important area of innovation, offering the opportunity to enhance patient experience without compromising diagnostic integrity. The main advantages and limitations of SLNB in melanoma are summarized in Table 3.

## 5. Conclusions

Sentinel lymph node biopsy (SLNB) has become an indispensable tool in the management of melanoma, revolutionizing the staging and treatment of this potentially deadly skin cancer. Since its introduction, SLNB has provided crucial prognostic information, allowing for more accurate risk stratification and personalized treatment strategies. By selectively identifying patients with occult lymph node metastases, SLNB has minimized the morbidity associated with complete lymph node dissection while still offering the potential for therapeutic benefit in some individuals. While SLNB remains the standard of care for clinically node-negative melanoma patients, ongoing research continues to refine the technique, explore alternative staging methods, and investigate the role of molecular profiling and immunotherapy in the management of patients with positive SLNs. Future directions in SLNB research will likely focus on optimizing lymphatic mapping and pathological evaluation techniques, identifying novel biomarkers for risk stratification, and integrating SLNB findings with emerging systemic therapies to further improve patient outcomes in melanoma.

## Figures and Tables

**Table 1:** Major Clinical Trials on SLNB in Melanoma.

Trial	Design	N (Patients)	Key Findings	Current Relevance
MSLT-I	Randomized: SLNB vs. observation	~2000	SLNB improves regional control, better identifies occult metastases; no OS benefit	Establishes SLNB as standard for staging
DeCOG-SLT	Randomized: CLND vs. observation after positive SLNB	~480	No difference in overall survival (OS)	Supports avoiding routine CLND
MSLT-II	Randomized: CLND vs. observation after positive SLNB	~1900	CLND does not improve OS; benefit limited to regional control	Practice change: routine CLND no longer recommended

**Table 2:** Current SLNB Indications According to Breslow and Guidelines (NCCN/ESMO).

Breslow/Ulceration	SLNB Recommendation
<0.8 mm, without ulceration	Not recommended
<0.8 mm with ulceration, or 0.8–1.0 mm	Consider SLNB
1.0–4.0 mm (intermediate)	Recommend SLNB
>4.0 mm	Recommend SLNB (prognosis and adjuvant therapy)

**Table 3:** Advantages and Limitations of SLNB in Melanoma.

Advantages	Limitations
Accurate nodal staging	Does not improve overall survival by itself
Robust prognostic information	Surgical risk (lymphedema, seroma, infection)
Selection of patients for adjuvant therapies	Costs and hospital resource requirements
Improves regional control	Debate on the necessity in all groups

## Data Availability

The original contributions presented in this study are included in the references. Further inquiries can be directed to the corresponding author(s).

## Abbreviations

The following abbreviations are used in this manuscript:

AL	Acral melanoma
ALM	Acral lentiginous melanoma
ARM	Anorectal melanoma
CI	Confidence interval
CjM	Conjunctival melanoma
CL	Complete lymphadenectomy
CLND	Complete lymph node dissection
CM	Cutaneous melanoma
DeCOG-SLT	German Cooperative Oncology Group selective node dissection trial
DFS	Disease-free survival
DM	Desmoplastic melanoma
ELND	Elective lymph node dissection
HR	Hazard ratio
MDM	Mixed desmoplastic melanoma
MelTUMPs	Melanocytic tumors of uncertain malignant potential
MSLT-I	Multicenter Selective Lymphadenectomy Trial I
MSLT-II	Multicenter Selective Lymphadenectomy Trial (MSLT-II)
NCCN	National Comprehensive Cancer Network
OS	Overall survival
PCR	Polymerase chain reaction
PD-1	Programmed cell death protein 1
PDM	Pure desmoplastic melanoma
PET/CT	Positron emission tomography/computed tomography
RFS	Recurrence-free survival
SEER	Surveillance, Epidemiology, and End Results
SLN	Sentinel lymph node(s)
SLNB	Sentinel lymph node biopsy
SPECT/CT	Single-photon emission computed tomography/computed tomography
TNM	Tumor, node, metastasis (staging system)
VM	Vulvar melanoma
WLE	Wide local excision

## References

[1] Bagaria S.P., Faries M.B., Morton D.L. (2010). Sentinel node biopsy in melanoma: Technical considerations of the procedure as performed at the john wayne cancer institute. J. Surg. Oncol..

[2] Brănişteanu D.E., Cozmin M., Porumb-Andrese E., Brănişteanu D., Toader M.P., Iosep D., Sinigur D., Brănişteanu C.I., Brănişteanu G., Porumb V. (2022). Sentinel Lymph Node Biopsy in Cutaneous Melanoma, a Clinical Point of View. Medicina.

[3] Murali R., Thompson J.F., Scolyer R.A. (2008). Sentinel Lymph Node Biopsy for Melanoma: Aspects of Pathologic Assessment. Future Oncol..

[4] Ross M.I., Gershenwald J.E. (2013). Sentinel lymph node biopsy for melanoma: A critical update for dermatologists after two decades of experience. Clin. Dermatol..

[5] Sadozai H., Gruber T., Hunger R.E., Schenk M. (2017). Recent Successes and Future Directions in Immunotherapy of Cutaneous Melanoma. Front. Immunol..

[6] van Esser S., Stapper G., van Diest P.J., van den Bosch M.A.A.J., Klaessens J.H.G.M., Mali W.P.T.M., Rinkes I.H.M.B., van Hillegersberg R. (2009). Ultrasound-Guided Laser-Induced Thermal Therapy for Small Palpable Invasive Breast Carcinomas: A Feasibility Study. Ann. Surg. Oncol..

[7] Thompson J.F. (2008). The Value of Sentinel Node Biopsy in Patients with Primary Cutaneous Melanoma. Dermatol. Surg..

[8] Harish K. (2005). Sentinel node biopsy: Concepts and current status. FBL.

[9] Young M.A. (2020). Lymphoscintigraphic Mapping in Melanoma and Breast Cancer. J. Nucl. Med. Technol..

[10] Leidenius M.H., Leppänen E.A., Krogerus L.A., Smitten K.A.v. (2004). The impact of radiopharmaceutical particle size on the visualization and identification of sentinel nodes in breast cancer. Nucl. Med. Commun..

[11] Dunson G.L., Thrall J.H., Stevenson J.S., Pinsky S.M. (1973). 99mTc Minicolloid for Radionuclide Lymphography. Radiology.

[12] Bianchi P., Villa G., Buffoni F., Agnese G., Gipponi M., Costa R., Maragliano C., Canavese G., Mariani G. (2000). Different Sites and Modes of Tracer Injection for Mapping the Sentinel Lymph Node in Patients with Breast Cancer. Tumori J..

[13] Nakamura Y., Otsuka F., Duc G.H.T. (2013). Sentinel Lymph Node Biopsy for Melanoma and Surgical Approach to Lymph Node Metastasis. Melanoma—From Early Detection to Treatment.

[14] Guo W., Zhang L., Ji J., Gao W., Liu J., Tong M. (2014). Evaluation of the benefit of using blue dye in addition to indocyanine green fluorescence for sentinel lymph node biopsy in patients with breast cancer. World J. Surg. Oncol..

[15] Uren R.F., Howman-Giles R., Chung D., Thompson J.F. (2015). Imaging Sentinel Lymph Nodes. Cancer J..

[16] Ganesh K., Massagué J. (2021). Targeting metastatic cancer. Nat. Med..

[17] Gershenwald J.E., Tseng C.-h., Thompson W., Mansfield P.F., Lee J.E., Bouvet M., Lee J., Ross M.I. (1998). Improved sentinel lymph node localization in patients with primary melanoma with the use of radiolabeled colloid. Surgery.

[18] Porter G.A., Ross M.I., Berman R.S., Sumner W.E., Lee J.E., Mansfield P.F., Gershenwald J.E. (2000). How many lymph nodes are enough during sentinel lymphadenectomy for primary melanoma?. Surgery.

[19] Faries M.B. (2024). Sentinel lymph nodes in melanoma: Necessary as ever for optimal treatment. Clin. Exp. Metastasis.

[20] Cassel C.K., Guest J.A. (2012). Choosing Wisely: Helping Physicians and Patients Make Smart Decisions About Their Care. JAMA.

[21] Cheng Z., Ma J., Yin L., Yu L., Yuan Z., Zhang B., Tian J., Du Y. (2023). Non-invasive molecular imaging for precision diagnosis of metastatic lymph nodes: Opportunities from preclinical to clinical applications. Eur. J. Nucl. Med. Mol. Imaging.

[22] Goyal A., Mansel R.E. (2004). Current status of sentinel lymph node biopsy in solid malignancies. World J. Surg. Oncol..

[23] Davids V., Kidson S.H., Hanekom G.S. (2003). Melanoma patient staging: Histopathological versus molecular evaluation of the sentinel node. Melanoma Res..

[24] van Diest P.J., Torrenga H., Meijer S., Meijer C.J.L.M. (2001). Pathologic analysis of sentinel lymph nodes. Semin. Surg. Oncol..

[25] Imam A. (1985). Application of Immunohistochemical Methods in the Diagnosis of Malignant Disease. Cancer Investig..

[26] Trotter M.J. (2011). Melanoma Margin Assessment. Clin. Lab. Med..

[27] Taylor C.R. (2009). Immunohistological Approach to Tumor Diagnosis. Oncology.

[28] Jeon B., Jung H.G., Lee S.W., Lee G., Shim J.H., Kim M.O., Kim B.J., Kim S.-H., Lee H., Lee S.W. (2022). Melanoma Detection by AFM Indentation of Histological Specimens. Diagnostics.

[29] Karimipour D.J., Lowe L., Su L., Hamilton T., Sondak V., Johnson T.M., Fullen D. (2004). Standard immunostains for melanoma in sentinel lymph node specimens: Which ones are most useful?. J. Am. Acad. Dermatol..

[30] Willis B.C., Johnson G., Wang J., Cohen C. (2015). SOX10: A useful marker for identifying metastatic melanoma in sentinel lymph nodes. Appl. Immunohistochem. Mol. Morphol..

[31] Cook M.G., Massi D., Szumera-Ciećkiewicz A., Van den Oord J., Blokx W., van Kempen L.C., Balamurugan T., Bosisio F., Koljenović S., Portelli F. (2019). An updated European Organisation for Research and Treatment of Cancer (EORTC) protocol for pathological evaluation of sentinel lymph nodes for melanoma. Eur. J. Cancer.

[32] Ordóñez N.G. (2014). Value of melanocytic-associated immunohistochemical markers in the diagnosis of malignant melanoma: A review and update. Hum. Pathol..

[33] Shidham V.B., Qi D.Y., Acker S., Kampalath B., Chang C.-C., George V., Komorowski R. (2001). Evaluation of Micrometastases in Sentinel Lymph Nodes of Cutaneous Melanoma: Higher Diagnostic Accuracy with Melan-A and MART-1 Compared with S-100 Protein and HMB-45. Am. J. Surg. Pathol..

[34] Ito T., Wada M., Nagae K., Nakano-Nakamura M., Nakahara T., Hagihara A., Furue M., Uchi H. (2015). Triple-marker PCR assay of sentinel lymph node as a prognostic factor in melanoma. J. Eur. Acad. Dermatol. Venereol..

[35] Blaheta H.-J., Ellwanger U., Schittek B., Maczey E., Breuninger H., Rassner G., Garbe C., Sotlar K., Thelen M.H., Bueltmann B. (2000). Examination of Regional Lymph Nodes by Sentinel Node Biopsy and Molecular Analysis Provides New Staging Facilities in Primary Cutaneous Melanoma. J. Investig. Dermatol..

[36] Voit C.A., Schäfer-Hesterberg G., Kron M., van Akkooi A.C.J., Rademaker J., Lukowsky A., Schoengen A., Schwürzer-Voit M., Sterry W., Krause M. (2008). Impact of Molecular Staging Methods in Primary Melanoma: Reverse-Transcriptase Polymerase Chain Reaction (RT-PCR) of Ultrasound-Guided Aspirate of the Sentinel Node Does Not Improve Diagnostic Accuracy, But RT-PCR of Peripheral Blood Does Predict Survival. J. Clin. Oncol..

[37] Lee J.H., Chen Y., Chan J.L., Qian Y.W., Goydos J.S. (2011). Molecular analysis of melanoma-induced sentinel lymph node immune dysfunction. Cancer Immunol. Immunother..

[38] Neves Rebello Alves L., Dummer Meira D., Poppe Merigueti L., Correia Casotti M., do Prado Ventorim D., Ferreira Figueiredo Almeida J., de Sousa V.P., Sant’ana M.C., da Cruz R.G.C., Louro L.S. (2023). Biomarkers in Breast Cancer: An Old Story with a New End. Genes.

[39] Dumitra T., Faries M.B. (2024). Melanoma sentinel lymph node biopsy in the modern era. Surg. Oncol..

[40] Cheng T.W., Hartsough E., Giubellino A. (2023). Sentinel lymph node assessment in melanoma: Current state and future directions. Histopathology.

[41] Neuhaus S.J., Clark M.A., Thomas J.M. (2004). Dr. Herbert Lumley Snow, MD, MRCS (1847–1930): The original champion of elective lymph node dissection in melanoma. Ann. Surg. Oncol..

[42] Morton D.L., Wen D.R., Wong J.H., Economou J.S., Cagle L.A., Storm F.K., Foshag L.J., Cochran A.J. (1992). Technical details of intraoperative lymphatic mapping for early stage melanoma. Arch. Surg..

[43] Morton D.L., Thompson J.F., Cochran A.J., Mozzillo N., Elashoff R., Essner R., Nieweg O.E., Roses D.F., Hoekstra H.J., Karakousis C.P. (2006). Sentinel-node biopsy or nodal observation in melanoma. N. Engl. J. Med..

[44] Valsecchi M.E., Silbermins D., de Rosa N., Wong S.L., Lyman G.H. (2011). Lymphatic mapping and sentinel lymph node biopsy in patients with melanoma: A meta-analysis. J. Clin. Oncol..

[45] Leiter U., Stadler R., Mauch C., Hohenberger W., Brockmeyer N., Berking C., Sunderkötter C., Kaatz M., Schulte K.-W., Lehmann P. (2016). Complete lymph node dissection versus no dissection in patients with sentinel lymph node biopsy positive melanoma (DeCOG-SLT): A multicentre, randomised, phase 3 trial. Lancet Oncol..

[46] Wong S.L., Faries M.B., Kennedy E.B., Agarwala S.S., Akhurst T.J., Ariyan C., Balch C.M., Berman B.S., Cochran A., Delman K.A. (2018). Sentinel Lymph Node Biopsy and Management of Regional Lymph Nodes in Melanoma: American Society of Clinical Oncology and Society of Surgical Oncology Clinical Practice Guideline Update. J. Clin. Oncol..

[47] Leiter U., Buettner P.G., Eigentler T.K., Bröcker E.B., Voit C., Gollnick H., Marsch W., Wollina U., Meier F., Garbe C. (2012). Hazard rates for recurrent and secondary cutaneous melanoma: An analysis of 33,384 patients in the German Central Malignant Melanoma Registry. J. Am. Acad. Dermatol..

[48] Australia M.I. (2025). Risk Prediction Tools. https://www.melanomarisk.org.au/.

[49] Center MSKC (2025). Melanoma Nomogram: Risk of Sentinel Lymph Node Metastasis. https://www.mskcc.org/nomograms/melanoma/sentinel_lymph_node_metastasis.

[50] Ollek S., Minkova S., Taqi K., Chen L., Martinka M., Davis N., Hamilton T., Stuart H. (2022). Population-based assessment of sentinel lymph node biopsy in the management of cutaneous melanoma. Can. J. Surg..

[51] Coit D.G., Thompson J.A., Albertini M.R., Barker C., Carson W.E., Contreras C., Daniels G.A., DiMaio D., Fields R.C., Fleming M.D. (2019). Cutaneous Melanoma, Version 2.2019, NCCN Clinical Practice Guidelines in Oncology. J. Natl. Compr. Cancer Netw..

[52] Swetter S.M., Tsao H., Bichakjian C.K., Curiel-Lewandrowski C., Elder D.E., Gershenwald J.E., Guild V., Grant-Kels J.M., Halpern A.C., Johnson T.M. (2019). Guidelines of care for the management of primary cutaneous melanoma. J. Am. Acad. Dermatol..

[53] Wright F.C., Souter L.H., Kellett S., Easson A., Murray C., Toye J., McCready D., Nessim C., Ghazarian D., Hong N.L. (2019). Primary excision margins, sentinel lymph node biopsy, and completion lymph node dissection in cutaneous melanoma: A clinical practice guideline. Curr. Oncol..

[54] Eggermont A.M., Chiarion-Sileni V., Grob J.J., Dummer R., Wolchok J.D., Schmidt H., Hamid O., Robert C., Ascierto P.A., Richards J.M. (2016). Prolonged Survival in Stage III Melanoma with Ipilimumab Adjuvant Therapy. N. Engl. J. Med..

[55] Long G.V., Hauschild A., Santinami M., Atkinson V., Mandalà M., Chiarion-Sileni V., Larkin J., Nyakas M., Dutriaux C., Haydon A. (2017). Adjuvant Dabrafenib plus Trametinib in Stage III BRAF-Mutated Melanoma. N. Engl. J. Med..

[56] Weber J., Mandala M., Del Vecchio M., Gogas H.J., Arance A.M., Cowey C.L., Dalle S., Schenker M., Chiarion-Sileni V., Marquez-Rodas I. (2017). Adjuvant Nivolumab versus Ipilimumab in Resected Stage III or IV Melanoma. N. Engl. J. Med..

[57] Eggermont A.M., Chiarion-Sileni V., Grob J.J., Dummer R., Wolchok J.D., Schmidt H., Hamid O., Robert C., A Ascierto P., Richards J.M. (2015). Adjuvant ipilimumab versus placebo after complete resection of high-risk stage III melanoma (EORTC 18071): A randomised, double-blind, phase 3 trial. Lancet Oncol..

[58] Hauschild A., Dummer R., Schadendorf D., Santinami M., Atkinson V., Mandalà M., Chiarion-Sileni V., Larkin J., Nyakas M., Dutriaux C. (2018). Longer Follow-Up Confirms Relapse-Free Survival Benefit with Adjuvant Dabrafenib Plus Trametinib in Patients with Resected. J. Clin. Oncol..

[59] Crystal J.S., Thompson J.F., Hyngstrom J., Caracò C., Zager J.S., Jahkola T., Bowles T.L., Pennacchioli E., Beitsch P.D., Multicenter Selective Lymphadenectomy Trials Study Group (2022). Therapeutic Value of Sentinel Lymph Node Biopsy in Patients with Melanoma: A Randomized Clinical Trial. JAMA Surg..

[60] Allard-Coutu A., Dobson V., Schmitz E., Shah H., Nessim C. (2023). The Evolution of the Sentinel Node Biopsy in Melanoma. Life.

[61] Murali R., Shaw H.M., Lai K., McCarthy S.W., Quinn M.J., Stretch J.R., Thompson J.F., Scolyer R.A. (2010). Prognostic factors in cutaneous desmoplastic melanoma: A study of 252 patients. Cancer.

[62] Conic R.Z., Ko J., Allam S.H., Atanaskova-Mesinkovska N., Kovalyshyn I., Bergfeld W., Gastman B.R. (2018). Mixed Versus Pure Variants of Desmoplastic Melanoma: The Cleveland Clinic Experience. Ann. Plast. Surg..

[63] Laeijendecker A.E., El Sharouni M.A., Sigurdsson V., van Diest P.J. (2020). Desmoplastic melanoma: The role of pure and mixed subtype in sentinel lymph node biopsy and survival. Cancer Med..

[64] Quinn M.J., Crotty K.A., Thompson J.F., Coates A.S., O’Brien C.J., McCarthy W.H. (1998). Desmoplastic and desmoplastic neurotropic melanoma: Experience with 280 patients. Cancer.

[65] Mohebati A., Ganly I., Busam K.J., Coit D., Kraus D.H., Shah J.P., Patel S.G. (2012). The role of sentinel lymph node biopsy in the management of head and neck desmoplastic melanoma. Ann. Surg. Oncol..

[66] Busam K.J., Mujumdar U., Hummer A.J., Nobrega J., Hawkins W.G., Coit D.G., Brady M.S. (2004). Cutaneous desmoplastic melanoma: Reappraisal of morphologic heterogeneity and prognostic factors. Am. J. Surg. Pathol..

[67] Hodson M., Feustel P., Davis L. (2022). Sentinel lymph node biopsy in desmoplastic melanoma—The percent desmoplastic component matters: A systematic review. J. Plast. Reconstr. Aesthet. Surg..

[68] Pawlik T.M., Ross M.I., Prieto V.G., Ballo M.T., Johnson M.M., Mansfield P.F., Lee J.E., Cormier J.N., Gershenwald J.E. (2006). Assessment of the role of sentinel lymph node biopsy for primary cutaneous desmoplastic melanoma. Cancer.

[69] Swetter S. (2020). NCCN Guidelines^®^ Insights: Melanoma: Cutaneous, Version 4.2020. http://medi-guide.meditool.cn/ymtpdf/ACC90A18-6CDF-9443-BF3F-E29394D495E8.pdf.

[70] Zell J.A., Cinar P., Mobasher M., Ziogas A., Meyskens F.L., Anton-Culver H. (2008). Survival for patients with invasive cutaneous melanoma among ethnic groups: The effects of socioeconomic status and treatment. J. Clin. Oncol..

[71] Board WCoTE (2023). Skin Tumors. WHO Classification of Tumours Series [Internet].

[72] Ryu G.W., Choi Y.D., Ryu Y.J., Lee J.B., Shin M.H., Yun S.J. (2021). Risk factors affecting the first metastasis of acral melanoma: Low- pigmentation independently predicts a first lung metastasis. J. Am. Acad. Dermatol..

[73] Ryu G.W., Choi Y.D., Jin S., Chung I.J., Shin M.H., Yun S.J. (2021). Volar location and degree of pigmentation are associated with poor survival and first metastasis pattern in acral melanoma. Pigment Cell Melanoma Res..

[74] Kunte C., Geimer T., Baumert J., Konz B., Volkenandt M., Flaig M., Ruzicka T., Berking C., Schmid-Wendtner M.-H. (2010). Prognostic factors associated with sentinel lymph node positivity and effect of sentinel status on survival: An analysis of 1049 patients with cutaneous melanoma. Melanoma Res..

[75] Gershenwald J.E., Thompson W., Mansfield P.F., Lee J.E., Colome M.I., Tseng C.H., Lee J.J., Balch C.M., Reintgen D.S., Ross M.I. (1999). Multi-institutional melanoma lymphatic mapping experience: The prognostic value of sentinel lymph node status in 612 stage I or II melanoma patients. J. Clin. Oncol..

[76] Kesmodel S.B., Karakousis G.C., Botbyl J.D., Canter R.J., Lewis R.T., Wahl P.M., Terhune K.P., Alavi A., Elder D.E., Ming M.E. (2005). Mitotic rate as a predictor of sentinel lymph node positivity in patients with thin melanomas. Ann. Surg. Oncol..

[77] Leong S.P., Kashani-Sabet M., Desmond R.A., Kim R.P., Nguyen D.H., Iwanaga K., Treseler P.A., Allen R.E., Morita E.T., Zhang Y. (2005). Clinical significance of occult metastatic melanoma in sentinel lymph nodes and other high-risk factors based on long-term follow-up. World J. Surg..

[78] Mraz-Gernhard S., Sagebiel R.W., Kashani-Sabet M., Miller J.R., Leong S.P. (1998). Prediction of sentinel lymph node micrometastasis by histological features in primary cutaneous malignant melanoma. Arch, Dermatol..

[79] Sondak V.K., Taylor J.M., Sabel M.S., Wang Y., Lowe L., Grover A.C., Chang A.E., Yahanda A.M., Moon J., Johnson T.M. (2004). Mitotic rate and younger age are predictors of sentinel lymph node positivity: Lessons learned from the generation of a probabilistic model. Ann. Surg. Oncol..

[80] Swetter S.M., Thompson J.A., Albertini M.R., Barker C.A., Baumgartner J., Boland G., Chmielowski B., DiMaio D., Durham A., Fields R.C. (2021). NCCN Guidelines^®^ Insights: Melanoma: Cutaneous, Version 2.2021. J. Natl. Compr. Cancrt Netw..

[81] Huang K., Fan J., Misra S. (2020). Acral Lentiginous Melanoma: Incidence and Survival in the United States, 2006–2015, an Analysis of the SEER Registry. J. Surg. Res..

[82] Ito T., Wada M., Nagae K., Nakano-Nakamura M., Nakahara T., Hagihara A., Furue M., Uchi H. (2015). Acral lentiginous melanoma: Who benefits from sentinel lymph node biopsy?. J. Am. Acad. Dermatol..

[83] Farooq M.S., Shafique N., Vargas G.M., Varey A.H.R., Lo S.N., Chu E.Y., Ming M.E., Miura J.T., Karakousis G.C. (2025). Utility of sentinel lymph node biopsy for acral lentiginous melanoma in the contemporary era: A national cohort analysis. J. Am. Acad. Dermatol..

[84] Curtin J.A., Fridlyand J., Kageshita T., Patel H.N., Busam K.J., Kutzner H., Cho K.-H., Aiba S., Bröcker E.-B., LeBoit P.E. (2005). Distinct sets of genetic alterations in melanoma. N. Engl. J. Med..

[85] Zebary A., Omholt K., Vassilaki I., Höiom V., Lindén D., Viberg L., Kanter-Lewensohn L., Johansson C.H., Hansson J. (2013). KIT, NRAS, BRAF and PTEN mutations in a sample of Swedish patients with acral lentiginous melanoma. J. Dermatol. Sci..

[86] Torres-Cabala C.A., Wang W.L., Trent J., Yang D., Chen S., Galbincea J., Kim K.B., Woodman S., Davies M., A Plaza J. (2009). Correlation between KIT expression and KIT mutation in melanoma: A study of 173 cases with emphasis on the acral-lentiginous/mucosal type. Mod. Pathol..

[87] Puig-Butillé J.A., Badenas C., Ogbah Z., Carrera C., Aguilera P., Malvehy J., Puig S. (2013). Genetic alterations in RAS-regulated pathway in acral lentiginous melanoma. Exp. Dermatol..

[88] Cohen Goldemberg D., de Melo A.C., de Melo Pino L.C., Thuler L.C.S. (2020). Epidemiological profile of mucosal melanoma in Brazil. Sci. Rep..

[89] Ciarrocchi A., Pietroletti R., Carlei F., Amicucci G. (2017). Extensive surgery and lymphadenectomy do not improve survival in primary melanoma of the anorectum: Results from analysis of a large database (SEER). Colorectal Dis..

[90] Perez D.R., Trakarnsanga A., Shia J., Nash G.M., Temple L.K., Paty P.B., Guillem J.G., Garcia-Aguilar J., Bello D., Ariyan C. (2013). Locoregional lymphadenectomy in the surgical management of anorectal melanoma. Ann. Surg. Oncol..

[91] Sanli Y., Turkmen C., Kurul S., Taş F., Mudun A., Cantez S. (2006). Sentinel lymph node biopsy for the staging of anal melanoma: Report of two cases. Ann. Nucl. Med..

[92] Olsha O., Mintz A., Gimon Z., Gold Deutch R., Rabin I., Halevy A., Reissman P. (2005). Anal melanoma in the era of sentinel lymph node mapping: A diagnostic and therapeutic challenge. Tech. Coloproctol..

[93] Mariolis-Sapsakos T., Malamitsi J., Yakoumakis E., Orfanos F. (2008). Is sentinel node mapping useful in anorectal melanoma?. Hell. J. Nucl. Med..

[94] Damin D.C., Rosito M.A., Spiro B.L. (2010). Long-term survival data on sentinel lymph node biopsy in anorectal melanoma. Tech. Coloproctol..

[95] Mistrangelo M., Picciotto F., Quaglino P., Marchese V., Lesca A., Senetta R., Leone N., Astrua C., Roccuzzo G., Orlando G. (2025). Feasibility and impact of sentinel lymph node biopsy in patients affected by ano-rectal melanoma. Tech. Coloproctol..

[96] Alimena S., Reid H., Bercow A., Feltmate C., St Laurent J., May T., Berkowitz R., Horowitz N., Davis M. (2025). Trends in sentinel lymph node evaluation for vulvar melanoma in the United States from 2012 to 2018. Gynecol. Oncol..

[97] Ragnarsson-Olding B., Johansson H., Rutqvist L.E., Ringborg U. (1993). Malignant melanoma of the vulva and vagina. Trends in incidence, age distribution, and long-term survival among 245 consecutive cases in Sweden 1960–1984. Cancer.

[98] Weinstock M.A. (1994). Malignant melanoma of the vulva and vagina in the United States: Patterns of incidence and population-based estimates of survival. Am. J. Obstet. Gynecol..

[99] Wohlmuth C., Wohlmuth-Wieser I., May T., Vicus D., Gien L.T., Laframboise S. (2020). Malignant Melanoma of the Vulva and Vagina: A US Population-Based Study of 1863 Patients. Am. J. Clin. Dermatol..

[100] Esmaeli B., Rubin M.L., Xu S., Goepfert R.P., Curry J.L., Prieto V.G., Ning J., Tetzlaff M.T. (2019). Greater Tumor Thickness, Ulceration, and Positive Sentinel Lymph Node Are Associated with Worse Prognosis in Patients with Conjunctival Melanoma: Implications for Future AJCC Classifications. Am. J. Surg. Pathol..

[101] Mor J.M., Rokohl A.C., Koch K.R., Heindl L.M. (2019). Sentinel lymph node biopsy in the management of conjunctival melanoma: Current insights. Clin. Ophthalmol..

[102] Hu D.N., Yu G., McCormick S.A., Finger P.T. (2008). Population-based incidence of conjunctival melanoma in various races and ethnic groups and comparison with other melanomas. Am. J. Ophthalmol..

[103] Wong J.R., Nanji A.A., Galor A., Karp C.L. (2014). Management of conjunctival malignant melanoma: A review and update. Expert Rev. Ophthalmol..

[104] Pfeiffer M.L., Savar A., Esmaeli B. (2013). Sentinel lymph node biopsy for eyelid and conjunctival tumors: What have we learned in the past decade?. Ophthalmic Plast. Reconstr. Surg..

[105] Cohen V.M., Tsimpida M., Hungerford J.L., Jan H., Cerio R., Moir G. (2013). Prospective study of sentinel lymph node biopsy for conjunctival melanoma. Br. J. Ophthalmol..

[106] Esmaeli B. (2019). Inclusion of Histologic Ulceration and Tumor Thickness in Future American Joint Committee on Cancer T Category Definitions for Conjunctival Melanoma. JAMA Ophthalmol..

[107] Brouwer N.J., Marinkovic M., van Duinen S.G., Bleeker J.C., Jager M.J., Luyten G.P.M. (2018). Treatment of conjunctival melanoma in a Dutch referral centre. Br. J. Ophthalmol..

[108] Peric B., Leiler S., Hawlina G., Jancar B., Snoj M., Perhavec A. (2021). Sentinel Node Biopsy for Conjunctival Melanoma; Single Centre Experience and Review of Current Literature. Cancer Control.

[109] Feller L., Khammissa R.A.G., Lemmer J. (2017). A Review of the Aetiopathogenesis and Clinical and Histopathological Features of Oral Mucosal Melanoma. Sci. World J..

[110] Nowowiejska J., Ordon A.J., Purpurowicz P., Argenziano G., Piccolo V. (2025). Many Faces of Melanoma: A Comparison Between Cutaneous, Mucosal, Acral, Nail, and Ocular Malignancy. Pigment Cell Melanoma Res..

[111] Leiter U., Eigentler T.K., Häfner H.M., Krimmel M., Uslu U., Keim U., Weide B., Breuninger H., Martus P., Garbe C. (2015). Sentinel Lymph Node Dissection in Head and Neck Melanoma has Prognostic Impact on Disease-Free and Overall Survival. Ann. Surg. Oncol..

[112] Wu Y., Wei D., Ren G., Guo W. (2023). Chemotherapy in combination with anti-PD-1 agents as adjuvant therapy for high-risk oral mucosal melanoma. J. Cancer Res. Clin. Oncol..

[113] Cerroni L., Barnhill R., Elder D., Gottlieb G., Heenan P., Kutzner H., LeBoit P.E., Mihm M.J., Rosai J., Kerl H. (2010). Melanocytic tumors of uncertain malignant potential: Results of a tutorial held at the XXIX Symposium of the International Society of Dermatopathology in Graz, October 2008. Am. J. Surg. Pathol..

[114] Magro C.M., Crowson A.N., Mihm M.C., Gupta K., Walker M.J., Solomon G. (2010). The dermal-based borderline melanocytic tumor: A categorical approach. J. Am. Acad. Dermatol..

[115] Varey A.H.R., Williams G.J., Lo S.N., Taing C.Y., Maurichi A., Santinami M., Scolyer R.A., Thompson J.F. (2023). Clinical management of melanocytic tumours of uncertain malignant potential (MelTUMPs), including melanocytomas: A systematic review and meta-analysis. J. Eur. Acad. Dermatol. Venereol..

[116] Kumar A.B., Jakub J.W., Lester S.C., Markovic S.N., Baum C.L. (2025). In-transit melanoma metastases: Evaluation and management for the dermatologist. J. Am. Acad. Dermatol..

[117] de Almiron A.C.-R., Serrano-Ortega S. (2012). Risk factors for in-transit metastasis in patients with cutaneous melanoma. Actas Dermo-Sifiliogr..

[118] Gyorki D.E. (2018). Management of In-Transit Melanoma: We Need Some High-Quality Data. J. Oncol. Pract..

[119] Dewar D.J., Powell B.W. (2003). Sentinel node biopsy in patients with in-transit recurrence of malignant melanoma. Br. J. Plast. Surg..

[120] Wainstein A.J.A., Cândido L.D., Drummond-Lage A.P. (2022). Sentinel Lymph Node Biopsy after Previous Radical Lymphadenectomy of the Same Lymph Node Basin. J. Invest. Surg..

[121] Stacker S.A., Williams S.P., Karnezis T., Shayan R., Fox S.B., Achen M.G. (2014). Lymphangiogenesis and lymphatic vessel remodelling in cancer. Nat. Rev. Cancer.

[122] Pastushenko I., Conejero C., Carapeto F.J. (2015). Lymphangiogenesis: Implications for diagnosis, treatment, and prognosis in patients with melanoma. Actas Dermo-Sifiliogr..

[123] Beasley G.M., Speicher P., Sharma K., Seigler H., Salama A., Mosca P., Tyler D.S. (2014). Efficacy of repeat sentinel lymph node biopsy in patients who develop recurrent melanoma. J. Am. Coll. Surg..

[124] Wada-Ohno M., Ito T., Furue M. (2019). Adjuvant Therapy for Melanoma. Curr. Treat. Options Oncol..

[125] Bartlett E.K., Peters M.G., Blair A., Etherington M.S., Elder D.E., Xu X.G., Guerry D., Ming M.E., Fraker D.L., Czerniecki B.J. (2016). Identification of Patients with Intermediate Thickness Melanoma at Low Risk for Sentinel Lymph Node Positivity. Ann. Surg. Oncol..

[126] deRosa N. (2025). Evolving Role of Sentinel Lymph Node Biopsy for Melanoma, Merkel Cell, and Squamous Cell Carcinoma. Surg. Clin. N. Am..

[127] Wong S.L., Brady M.S., Busam K.J., Coit D.G. (2006). Results of Sentinel Lymph Node Biopsy in Patients with Thin Melanoma. Ann. Surg. Oncol..

[128] Durham A.B., Schwartz J.L., Lowe L., Zhao L., Johnson A.G., Harms K.L., Bichakjian C.K., Orsini A.P., McLean S.A., Bradford C.R. (2017). The natural history of thin melanoma and the utility of sentinel lymph node biopsy. J. Surg. Oncol..

[129] Andtbacka R.H.I., Gershenwald J.E. (2009). Role of Sentinel Lymph Node Biopsy in Patients with Thin Melanoma. J. Natl. Compr. Cancer Netw..

[130] Shannon A.B., Sharon C.E., Straker R.J., Carr M.J., Sinnamon A.J., Bogatch K., Thaler A., Kelly N., Vetto J.T., Fowler G. (2023). Sentinel lymph node biopsy in patients with T1a cutaneous malignant melanoma: A multicenter cohort study. J. Am. Acad. Dermatol..

[131] Tejera-Vaquerizo A., Boada A., Ribero S., Puig S., Paradela S., Moreno-Ramírez D., Cañueto J., de Unamuno-Bustos B., Brinca A., Descalzo-Gallego M.A. (2021). Sentinel Lymph Node Biopsy vs. Observation in Thin Melanoma: A Multicenter Propensity Score Matching Study. J. Clin. Med..

[132] Wright B.E., Scheri R.P., Ye X., Faries M.B., Turner R.R., Essner R., Morton D.L. (2008). Importance of Sentinel Lymph Node Biopsy in Patients with Thin Melanoma. Arch. Surg..

[133] Hughes B., Leijte J., Shabbir M., Watkin N., Horenblas S. (2009). Non-invasive and minimally invasive staging of regional lymph nodes in penile cancer. World J. Urol..

[134] Harisinghani M.G., Weissleder R. (2004). Sensitive, Noninvasive Detection of Lymph Node Metastases. PLoS Med..

[135] Rizzetto G., Lucarini G., De Simoni E., Molinelli E., Mattioli-Belmonte M., Offidani A., Simonetti O. (2022). Tissue Biomarkers Predicting Lymph Node Status in Cutaneous Melanoma. Int. J. Mol. Sci..

[136] Venturi F., Magnaterra E., Scotti B., Ferracin M., Dika E. (2025). Predictive Factors for Sentinel Lymph Node Positivity in Melanoma Patients—The Role of Liquid Biopsy, MicroRNA and Gene Expression Profile Panels. Cancers.

[137] Kangas-Dick A.W., Greenbaum A., Gall V., Groisberg R., Mehnert J., Chen C., Moore D.F., Berger A.C., Koshenkov V. (2021). Evaluation of a Gene Expression Profiling Assay in Primary Cutaneous Melanoma. Ann. Surg. Oncol..

[138] Kött J., Zimmermann N., Zell T., Rünger A., Heidrich I., Geidel G., Smit D.J., Hansen I., Abeck F., Schadendorf D. (2024). Sentinel lymph node risk prognostication in primary cutaneous melanoma through tissue-based profiling, potentially redefining the need for sentinel lymph node biopsy. Eur. J. Cancer.

[139] Bartlett E.K., O’Donoghue C., Boland G., Bowles T., Delman K.A., Hieken T.J., Moncrieff M., Wong S., White R.L., Karakousis G. (2025). Society of Surgical Oncology Consensus Statement: Assessing the Evidence for and Utility of Gene Expression Profiling of Primary Cutaneous Melanoma. Ann. Surg. Oncol..

[140] Kimbrough C.W., Egger M.E., McMasters K.M., Stromberg A.J., Martin R.C.G., Philips P., Scoggins C.R. (2016). Molecular Staging of Sentinel Lymph Nodes Identifies Melanoma Patients at Increased Risk of Nodal Recurrence. J. Am. Coll. Surg..

[141] Akbani R., Akdemir K.C., Aksoy B.A., Albert M., Ally A., Amin S.B., Arachchi H., Arora A., Auman J.T., Ayala B. (2015). Genomic Classification of Cutaneous Melanoma. Cell.

[142] Torphy R.J., Friedman C., Ho F., Leonard L.D., Thieu D., Lewis K.D., Medina T.M., Robinson W.A., Gonzalez R.C., Stewart C.L. (2022). Adjuvant Therapy for Stage III Melanoma Without Immediate Completion Lymph Node Dissection. Ann. Surg. Oncol..

[143] Farrow N.E., Raman V., Williams T.P., Nguyen K.Y., Tyler D.S., Beasley G.M. (2020). Adjuvant Therapy is Effective for Melanoma Patients with a Positive Sentinel Lymph Node Biopsy Who Forego Completion Lymphadenectomy. Ann. Surg. Oncol..

[144] Herb J.N., Dunham L.N., Ollila D.W., Stitzenberg K.B., Meyers M.O. (2020). Use of Completion Lymph Node Dissection for Sentinel Lymph Node-Positive Melanoma. J. Am. Coll. Surg..

[145] Alliance M.R. Sentinel Lymph Node Biopsy in Melanoma. https://www.curemelanoma.org/patient-eng/melanoma-treatment/options/sentinel-lymph-node-biopsy.

[146] Yamashita K., Shimizu K. (2008). Video-assisted breast surgery and sentinel lymph node biopsy guided by three-dimensional computed tomographic lymphography. Surg. Endosc..

[147] Sorrentino L., Sartani A., Pietropaolo G., Bossi D., Mazzucchelli S., Truffi M., Foschi D., Corsi F. (2018). A Novel Indocyanine Green Fluorescence-Guided Video-Assisted Technique for Sentinel Node Biopsy in Breast Cancer. World J. Surg..

[148] Mok C.W., Hing J.X.J., Shetty S.S., Tan S.-M. (2020). Endoscopic-assisted ICG (EASI) technique for sentinel lymph node biopsy in breast cancer. Mini-Invasive Surg..

[149] Stoffels I., Dissemond J., Pöppel T., Schadendorf D., Klode J. (2015). Intraoperative Fluorescence Imaging for Sentinel Lymph Node Detection: Prospective Clinical Trial to Compare the Usefulness of Indocyanine Green vs Technetium Tc 99m for Identification of Sentinel Lymph Nodes. JAMA Surg..

[150] Stoffels I., Jansen P., Petri M., Goerdt L., Brinker T.J., Griewank K.G., Poeppel T.D., Schadendorf D., Klode J. (2019). Assessment of Nonradioactive Multispectral Optoacoustic Tomographic Imaging with Conventional Lymphoscintigraphic Imaging for Sentinel Lymph Node Biopsy in Melanoma. JAMA Netw. Open..

[151] Hotchkies A., Saiyed S., Palaniappan S., Koroma P., Sarsam T., Falls D., Hanif S., Rahman S., ElBatawy A. (2024). Efficacy of indocyanine green fluoroscopy for sentinel node biopsy in head and neck melanoma: A systematic review and meta-analysis. Br. J. Oral Maxillofac. Surg..

[152] Lafreniere A.S., Shine J.J., Nicholas C.R., Temple-Oberle C.F. (2021). The use of indocyanine green and near-infrared fluorescence imaging to assist sentinel lymph node biopsy in cutaneous melanoma: A systematic review. Eur. J. Surg. Oncol..

[153] Wölffer M., Liechti R., Constantinescu M., Lese I., Zubler C. (2024). Sentinel Lymph Node Detection in Cutaneous Melanoma Using Indocyanine Green-Based Near-Infrared Fluorescence Imaging: A Systematic Review and Meta-Analysis. Cancers.

